# The Science Behind Competition and Winning in Athletics: Using World-Level Competition Data to Explore Pacing and Tactics

**DOI:** 10.3389/fspor.2019.00011

**Published:** 2019-08-08

**Authors:** Florentina J. Hettinga, Andrew M. Edwards, Brian Hanley

**Affiliations:** ^1^Department of Sport, Exercise and Rehabilitation, Faculty of Health and Life Sciences, Northumbria University, Newcastle upon Tyne, United Kingdom; ^2^School of Human and Life Sciences, Canterbury Christ Church University, Canterbury, United Kingdom; ^3^Carnegie School of Sport, Leeds Beckett University, Leeds, United Kingdom

**Keywords:** athletics, pacing, race performance, running, tactics

## Abstract

The purpose of this study was to examine whether World Championship and Olympic medallist endurance athletes pace similarly to their race opponents, where and when critical differences in intra-race pacing occur, and the tactical strategies employed to optimally manage energy resources. We analyzed pacing and tactics across the 800, 1,500, 5,000, 10,000 m, marathon and racewalk events, providing a broad overview for optimal preparation for racing and pacing. Official electronic splits from men's (*n* = 275 performances) and women's (*n* = 232 performances) distance races between 2013 and 2017 were analyzed. Athletes were grouped for the purposes of analysis and comparison. For the 800 m, these groups were the medalists and those finishing 4th to 8th (“Top 8”). For the 1,500 m, the medalists and Top 8 were joined by those finishing 9th to 12th (“Top 12”), whereas for all other races, the Top 15 were analyzed (those finishing 9th to 15th). One-way repeated measures analysis of variance was conducted on the segment speeds (*p* < 0.05), with effect sizes for differences calculated using Cohen's *d*. Positive pacing profiles were common to most 800 m athletes, whereas negative pacing was more common over longer distances. In the 1,500 m, male medalists separated from their rivals in the last 100 m, whereas for women it was after 1,200 m. Similarly, over 5,000 m, male medalists separated from the slowest pack members later (4,200 m; 84% of duration) than women (2,500 m; 50% of duration). In the 10,000 m race, the effect was very pronounced with men packing until 8,000 m, with the Top 8 athletes only dropped at 9,600 m (96% of duration). For women, the slowest pack begin to run slower at only 1,700 m, with the Top 8 finishers dropped at 5,300 m (53% of duration). Such profiles and patterns were seen across all events. It is possible the earlier separation in pacing for women between the medalists and the other runners was because of tactical racing factors such as an early realization of being unable to sustain the required speed, or perhaps because of greater variation in performance abilities.

## Introduction

Every two years, the International Association of Athletics Federations (IAAF) World Championships are held. This provides us with an ideal opportunity to provide up-to-date scientific knowledge on aspects relevant to performance, competition and winning, which will clearly be relevant to athletes and coaches taking part in these and other similar championships. Specific problems encountered by athletes in competition, particularly in the longer race events, relate to distributing available energy over a race (i.e., pacing) and how to optimally engage in interpersonal competition (i.e., tactics). Both aspects have been found to be decisive factors in athlete performance (Konings and Hettinga, 2018a). Athletes are required to decide continuously about how and when to invest their limited energy resources over time to win the race (Edwards and Polman, 2013; Smits et al., 2014) not only when racing alone, but also when racing against other competitors (Hettinga et al., 2017), as occurs in IAAF World Championships. When in competition, opponents and other environmental factors influence motivation, attentional focus (perception), the ability to tolerate fatigue and pain, positioning, drafting, falls risk, and collective behavior, and hence race performance cannot be examined in isolation. Therefore, it is important to analyze real-life competitive events, and sport environments are nowadays ideally suited to provide us with large datasets on how humans behave, decide and perform under physically challenging circumstances (Hettinga, 2018). In Olympic and World Championship short track speedskating, this has already been shown to be successful and resulted in a better understanding of the impact of tactics (Konings et al., 2016a; Noorbergen et al., 2016; Konings and Hettinga, 2018b), preceding qualifying races (Konings and Hettinga, 2018c), and different competitive environments (Konings and Hettinga, 2018d) on pacing and performance.

In athletics, several studies have focused on world record performances, paced races in Diamond League meets, and the mass fields in city marathons (Deaner et al., 2015; Díaz et al., 2018; Filipas et al., 2018). By contrast, the first studies analyzing pacing and tactics in both men and women using official timing split data across multiple IAAF World Championships have only been completed recently (Hanley, 2013, 2016; Filipas et al., 2019; Hanley et al., 2019). The results confirmed the potential of rigorous analysis of competition data across endurance races, finding that successful middle-distance athletes generally separated themselves from slower athletes in the final 200 m, not by speeding up, but by avoiding slowing compared with competitors. Pacing variability was high compared with world records and longer distance events, especially in the finals, showing that athletes must cope with varied pace and surges. It was also recently found that World Championship middle-distance finalists were racers, rather than pacers (Hanley and Hettinga, 2018) and approached each round with a strategy of winning, rather than necessarily focusing on optimizing energy conservation (Brown, 2005).

Although recent 800 and 1,500 m world-class race data have previously been explored in quite some detail (Hanley and Hettinga, 2018; Hanley et al., 2019), for the longer distances of 5,000, 10,000 m, marathon and 20 and 50 km racewalks, the most recent championship performances have not yet been included or have relied on low resolution data (e.g., every 1,000 m in the track events, and every 5 km in the road events). For the 5,000 and 10,000 m, 1,000-m split analyses have been conducted up until 2017 (Filipas et al., 2019), but 100-m splits are available for the very recent Olympic Games and World Championships of 2013, 2016, and 2017, which could provide additional insights into variability of race performances as completed for the middle-distances (Hanley and Hettinga, 2018; Hanley et al., 2019). For the marathon, data have been analyzed using 5-km split data from 2011 up until 2015 (Hanley, 2016). Race data from 2016 and 2017 are now available and can be included for analysis to provide more insights into developments of marathon performance throughout the last two decades, and more specifically, in relation to the current crop of world-class distance athletes. This point is particularly pertinent for coaches as pacing can be strongly dictated by the presence of a single contemporary, uniquely talented athlete (Sandford et al., 2018). Lastly, the 20 and 50 km racewalks have been analyzed using 5-km split times including data from 1999 to 2011 (Hanley, 2013), but more recent data are available to us, and with higher resolutions of 1-km or 2-km splits. The ban on Russian athletes competing (with the exception of Authorized Neutral Athletes) since 2015 has had its biggest effect on racewalking as, notwithstanding some subsequent disqualifications, Russian athletes finished first in 11 of the 15 racewalk events held at the World Championships from 2005 to 2013, and changes in pacing profiles might have occurred since the previous analyses. Most notably, no women's data have been analyzed for the 50 km racewalk before, as this event was first held at a global championship in 2017. High-resolution 1-km split data from this event were made available to us by the official timing company to analyze and will provide new insights into women's athletics.

In the current paper, we will bring together all up-to-date available data from Olympic and World Championship finals in the 800, 1,500, 5,000, 10,000 m, marathon, 20 and 50 km racewalk to explore the science behind competition and winning in world-level championships. We will include recent data in our analyses to explore pacing and tactics of both men and women athletes, and we will discuss these in the specific context of global championships to provide athletes and coaches with valuable insights and advice to prepare for competition.

## Materials and Methods

Official electronic finishing and split times for the men's and women's middle-distance, long-distance and racewalk finals at the IAAF World Championships in 2013 and 2017, as well as the Olympic Games in 2016, were obtained from the open-access IAAF website (IAAF, 2019), published results (Almeida, 2016), and directly from the official timing company for the IAAF World Championships, SEIKO (with permission from the IAAF). Split data for each 100-m segment of the 800, 1,500, 5,000 and 10,000 m finals were analyzed; for the 800 m, high-resolution split data from the 2016 Olympic Games were not available. For the marathon, race split times were obtained for each 5 km, halfway (21.098 km) and the finish (42.195 km). For convenience, the final segment distance in the marathon is described in this study as 2.2 km. For the 20 km racewalks, split data for each 2 km loop have been analyzed, whereas for the 50 km racewalks, split data for each 5-km segment have been analyzed. The women's 50 km racewalk was first held in 2017, with its late addition meaning very few athletes (*n* = 7) took part. Because we were able to obtain 1-km splits for each racewalk event at the 2017 IAAF World Championships courtesy of SEIKO and the IAAF, we have also included analyses of these specific races at this higher 1-km resolution. All split data were recorded using transponders carried by the athletes (usually as part of their number bib) that used radio-frequency identification (RFID); finishing times were recorded using official electronic timing devices that were accurate to 1/1000 s (IAAF, 2015).

In each race, athletes were grouped for the purposes of analysis and comparison. For the 800 m, these groups were the top three finishers (“medalists”) and those finishing 4th to 8th (“Top 8”). For the 1,500 m, the medalists and top 8 groups were joined by those finishing 9th to 12th (“Top 12”), whereas for all other races, the groups were the medalists, Top 8 and Top 15 (i.e., those finishing 9th to 15th). Athletes who have been subsequently disqualified since the championships (e.g., for doping offenses) have not been included. Hence, an athlete finishing 9th behind a subsequently disqualified athlete, for example, has been considered to have finished 8th. If split data were missing for an athlete at any distance, that athlete has not been included in the study (i.e., one woman finishing 15th in the 10,000 m and one man finishing 15th in the marathon). Overall, 275 men's and 232 women's performances were analyzed, which included performances by 102 athletes who competed in more than one championship.

### Data Analysis

The study was designed as observational research in describing pacing profiles in recent world-class endurance championships in athletics. Athletes' split times were used to calculate mean speed during each 100-m, 1-km, 2-km, or 5-km segment (as appropriate) before the given split (e.g., 0–100 m was termed the 100-m segment). In the marathon, the split time for halfway (21.098 km) was also recorded. A positive split was considered to occur when an athlete ran the second half of the race in a longer time than the first, and a negative split occurred when the first half was longer (Abbiss and Laursen, 2008). To calculate whether athletes ran a positive or negative split in the 1,500 m, the 700–800-m split time was divided by two and this halved time added to the first and second 700-m segments; for all other events, halfway data were available for this purpose. To allow easier comparison between events, all running and racewalking speeds are presented as km/h. Pace variability was measured using coefficient of variation of all segments, calculated as a percentage (CV%) for each athlete's performance using the mean and standard deviation (SD) of all their segment speeds.

### Statistics

One-way repeated measures analysis of variance (ANOVA) was conducted on the segment speeds, with repeated contrast tests conducted to identify changes between successive segments (Field, 2009). Greenhouse-Geisser corrections were used if Mauchly's test for sphericity was violated. Segment speeds between the two groups in the 800 m events were compared using independent *t*-tests, whereas one-way ANOVA with Tukey's *post-hoc* tests were conducted to compare mean segment speeds when three groups were analyzed (Field, 2009). To compare men's and women's pacing profiles, individuals' speeds for each section were expressed as a percentage of their mean speed for the whole race, and grouped together: medalists and Top 8 in the 800 m; medalists, Top 8 and Top 12 in the 1,500 m; and medalists, Top 8 and Top 15 in all other events. These percentage data were arcsine transformed for the purposes of statistical analysis (Hanley, 2018) and compared using independent *t*-tests. Statistical significance was accepted as *p* < 0.05. 95% confidence intervals (95% CI) were also calculated (Field, 2009). Effect sizes for differences between successive segments and between groups for each segment were calculated using Cohen's *d* (Cohen, 1988), rounded to two decimal places and considered to be either trivial (*d* < 0.20), small (0.21–0.60), moderate (0.61–1.20), large (1.21–2.00), or very large (2.01–4.00) (Hopkins et al., 2009). In all figures and tables (and the text below), differences between successive splits have been annotated only when the effect size was moderate or larger (*d* ≥ 0.61) and the 95% CI did not cross zero. The distances at which differences were found between groups for segment speeds are also shown.

## Results

**Figure 1:**
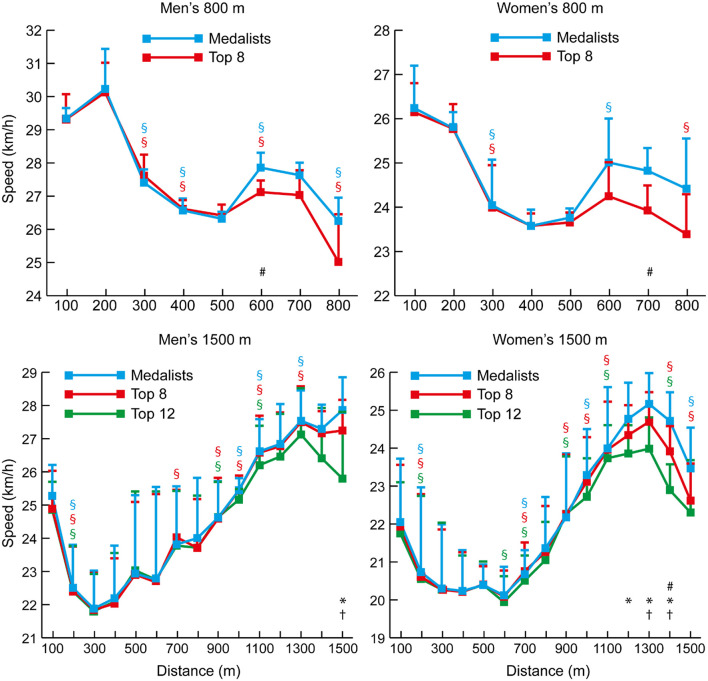
The mean (+ SD) 100-m segment speed for each group of men and women 800 and 1,500 m athletes. Differences between successive segments (*p* < 0.05, *d* ≥ 0.61) are represented by § (blue for medalists, red for Top 8, and green for Top 12). Differences in segment speed between groups (*p* < 0.05, *d* ≥ 0.61) have been annotated for medalists vs. Top 8 (#), medalists vs. Top 12 (1,500 m only) (^*^), and Top 8 vs. Top 12 (1,500 m only) (†).

**Figure 2:**
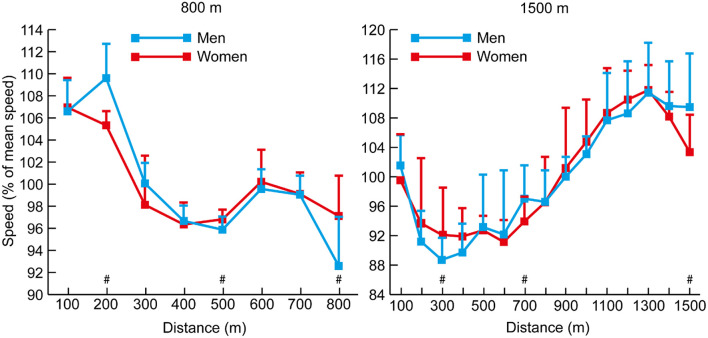
The mean (+ SD) section speed expressed as a percentage of mean speed for all men and women in the 800 and 1,500 m events. Differences in segment speed percentage between men and women (*p* < 0.05, *d* ≥ 0.61) have been annotated (#).

The mean finishing times for each group in each race demonstrated the world-class standard of the competitors analyzed (Table 1). There was higher variability in pace in the shorter events of the 800, 1,500, and 5,000 m (Table 2); the mean speeds for each 100-m split for each group of 800 and 1,500 m athletes (Figure 1) highlight the considerable variation in speed in each event. In the 800 m, the percentage differences in running time between the first and second halves (Table 3) show that it is the only distance with consistently slower second halves. In the men's event, all athletes ran a positive split, whereas in the women's event, two of the six medalists ran negative splits; all other 800 m women ran positive splits. By contrast, in the 1,500 m events, all men and women ran negative splits. The mean 100-m segment speed percentages for all men and all women analyzed in the 800 m and 1,500 m (Figure 2) showed that men had relatively faster splits in the first quarter of the 800 m (*p* < 0.001, *d* = 1.64, 95% CI = 2.56–7.00), but slower ones in the 1,500 m (*p* = 0.001, *d* = 0.73, 95% CI = 2.06–7.58). Men were relatively slower in the last 100 m of the 800 m (*p* = 0.016, *d* = 1.12, 95% CI = 1.31–11.91), but were faster in the same section in the 1,500 m (*p* = 0.001, *d* = 0.99, 95% CI = 4.16–14.26).

**Table 1:** Mean (±SD) finishing times for each group of men and women athletes in each event.

	**Medalists**	**Top 8**	**Top 12/15**
**MEN**			
800 m	1:44.24(±0.80)	1:45.48(±1.04)	
1,500 m	3:40.26(±7.48)	3:41.19(±7.06)	3:43.20(±6.59)
5,000 m	13:21.37(±13.40)	13:24.30(±12.46)	13:35.25(±15.79)
10,000 m	27:05.96(±13.94)	27:12.94(±13.44)	27:30.80(±12.89)
Marathon	2:09:42(±0:39)	2:11:22(±0:33)	2:13:24(±0:55)
20 km racewalk	1:19:58(±1:13)	1:20:38(±1:16)	1:21:33(±1:18)
50 km racewalk	3:39:47(±2:42)	3:43:26(±1:46)	3:47:46(±1:56)
**WOMEN**			
800 m	1:56.80(±1.10)	1:58.59(±0.97)	
1,500 m	4:05.28(±3.52)	4:07.18(±3.80)	4:09.86(±4.05)
5,000 m	14:40.02(±9.55)	14:57.07(±9.05)	15:21.83(±20.19)
10,000 m	30:21.17(±40.61)	30:54.67(±33.05)	31:50.09(±30.72)
Marathon	2:26:00(±1:28)	2:29:12(±3:38)	2:32:09(±3:59)
20 km racewalk	1:27:40(±1:03)	1:29:02(±0:44)	1:30:47(±0:49)

*Times are presented as min:s for the 800, 1,500, 5,000 and 10,000 m, and as h:min:s in the marathon and racewalks*.

**Table 2:** Mean (±SD) CV% for each group of men and women athletes in each event.

	**Medalists**	**Top 8**	**Top 12/15**
**MEN**			
800 m	5.5 (±0.9)	6.4 (±1.6)	
1,500 m	9.2 (±4.5)	9.0 (±4.2)	8.5 (±4.4)
5,000 m	8.5 (±1.7)	7.5 (±2.2)	5.9 (±1.9)
10,000 m	5.1 (±0.5)	4.1 (±0.7)	3.2 (±0.6)
Marathon	2.8 (±1.1)	2.6 (±1.0)	3.5 (±2.2)
20 km racewalk	1.7 (±0.7)	1.4 (±0.5)	1.9 (±0.9)
50 km racewalk	1.6 (±0.7)	1.7 (±0.9)	2.5 (±1.5)
**WOMEN**			
800 m	4.6 (±1.6)	4.9 (±1.2)	
1,500 m	9.7 (±3.0)	8.8 (±3.1)	8.0 (±2.7)
5,000 m	6.9 (±1.6)	5.7 (±1.5)	4.5 (±1.5)
10,000 m	4.9 (±1.8)	4.1 (±1.6)	3.8 (±1.2)
Marathon	2.6 (±1.1)	3.0 (±1.6)	2.9 (±1.3)
20 km racewalk	3.7 (±0.9)	2.6 (±0.7)	1.8 (±0.8)

**Table 3:** Mean (±SD) split percentages for each group of men and women athletes in each event based on halfway split and finishing times.

	**Medalists**	**Top 8**	**Top 12/15**
**MEN**			
800 m	+4.6(±1.3)	+7.0(±2.0)	
1,500 m	−14.9(±9.1)	−14.7(±8.3)	−12.8(±9.6)
5,000 m	−8.7(±4.5)	−8.1(±4.6)	−5.1(±5.2)
10,000 m	−3.4(±1.4)	−2.6(±1.4)	−0.7(±1.4)
Marathon	−2.2(±1.7)	+0.4(±1.2)	+2.7(±1.6)
20 km racewalk	−1.9(±1.3)	−0.3(±1.5)	+1.2(±1.8)
50 km racewalk	−2.0(±1.2)	+0.2(±2.1)	+1.5(±2.9)
**WOMEN**			
800 m	+1.4(±3.0)	+4.1(±3.1)	
1,500 m	−15.1(±8.8)	−13.6(±8.3)	−12.1(±8.2)
5,000 m	−7.6(±2.8)	−5.0(±2.6)	−0.6(±2.8)
10,000 m	−3.3(±3.5)	−0.4(±4.1)	+0.4(±3.4)
Marathon	−1.7(±2.0)	+0.5(±4.3)	+2.2(±2.8)
20 km racewalk	−5.7(±1.5)	−2.3(±2.6)	+0.0(±1.5)

*Positive values indicate positive pacing (i.e., the athletes slowed in the second half) and are indicated with a + sign for emphasis, and negative values indicate negative pacing*.

**Figure 3:**
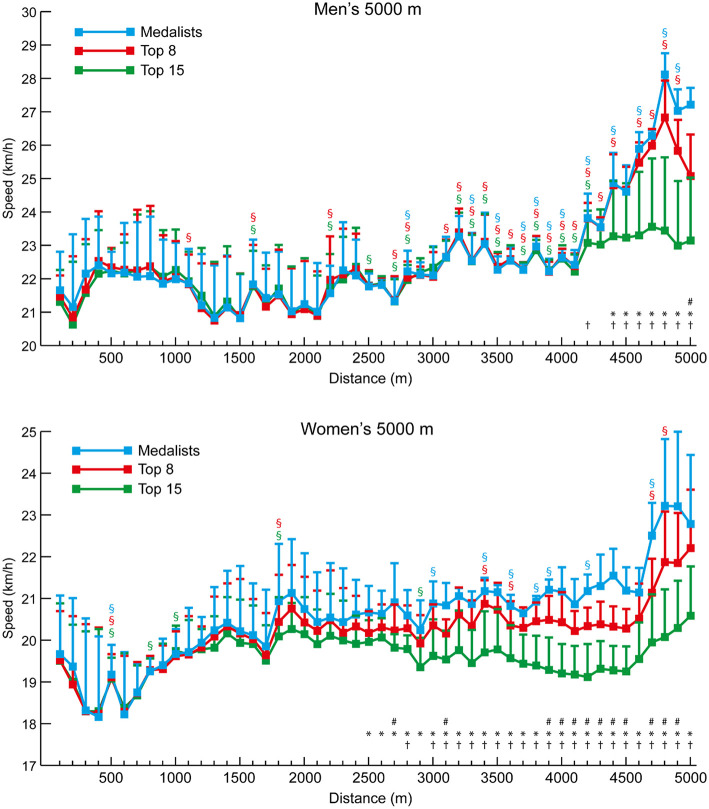
The mean (+ SD) 100-m segment speed for each group of men and women 5,000 m athletes. Differences between successive segments (*p* < 0.05, *d* ≥ 0.61) are represented by § (blue for medalists, red for Top 8, and green for Top 15). Differences in segment speed between groups (*p* < 0.05, *d* ≥ 0.61) have been annotated for medalists vs. Top 8 (#), medalists vs. Top 15 (^*^), and Top 8 vs. Top 15 (†).

**Figure 4:**
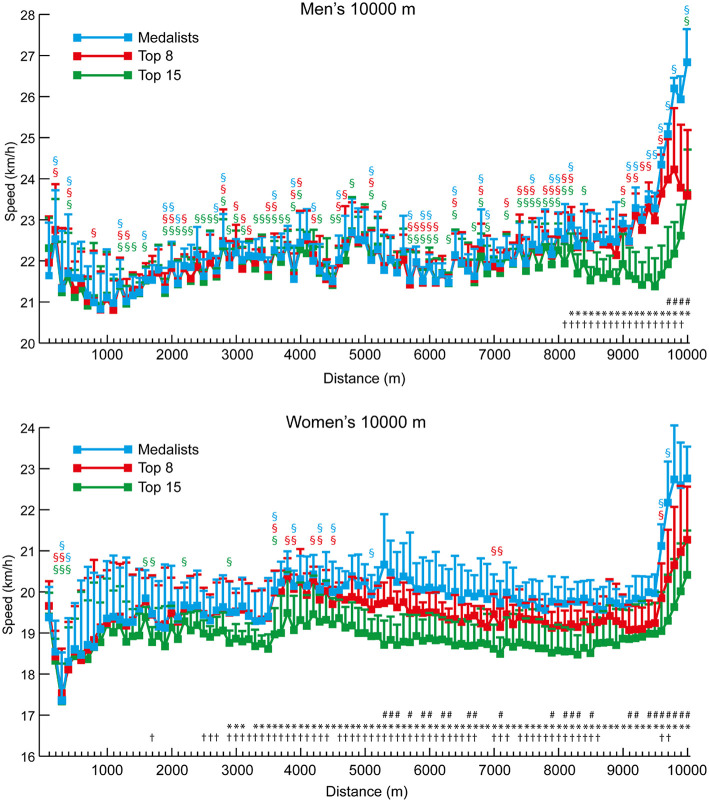
The mean (+ SD) 100-m segment speed for each group of men and women 10,000 m athletes. Differences between successive segments (*p* < 0.05, *d* ≥ 0.61) are represented by § (blue for medalists, red for Top 8, and green for Top 15). Differences in segment speed between groups (*p* < 0.05, *d* ≥ 0.61) have been annotated for medalists vs. Top 8 (#), medalists vs. Top 15 (^*^), and Top 8 vs. Top 15 (†).

**Figure 5:**
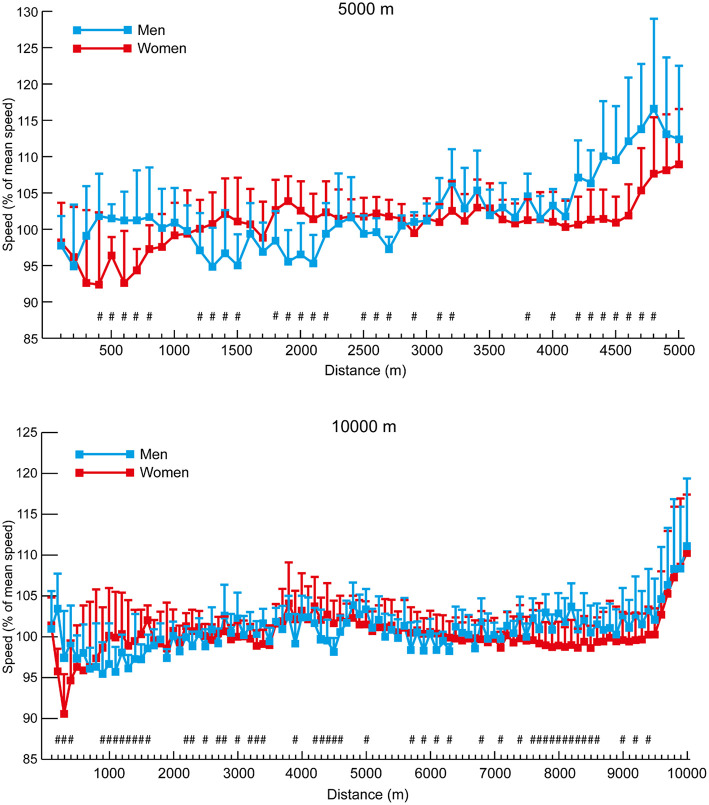
The mean (+ SD) section speed expressed as a percentage of mean speed for all men and women in the 5,000 and 10,000 m events. Differences in segment speed percentage between men and women (*p* < 0.05, *d* ≥ 0.61) have been annotated (#).

In general, the pacing profiles of the 5,000 m and 10,000 m races showed typical championship patterns of slightly varying pace until the final laps (Figures 3, 4, respectively). This resulted in all medalists and Top 8 athletes in the men's and women's 5,000 m races running negative splits, although the fact that four of the 21 men and eight of the 20 women in the Top 15 groups did not shows the inability of these lower-finishing athletes to maintain pace with faster athletes. Similarly, all of the medalists and Top 8 athletes in the men's 10,000 m ran negative splits, but seven of the 21 men in the Top 15 group ran positive splits. Likewise, eight of the nine medalists in the women's 10,000 m ran negative splits, as well as seven of the 15 Top 8 athletes, and 13 of the 20 women who were included in the Top 15 group ran positive splits. The mean 100-m segment speed percentages for all men and all women analyzed in the 5,000 m and 10,000 m (Figure 5) show that there were occasional differences in the pacing profiles adopted, with men noticeably finishing relatively faster in the 5,000 m (4,200–4,800 m: *p* ≤ 0.003, *d* = 0.89–1.57). It was also noticeable that the separation of groups occurred earlier in the women's races (women medalists vs. Top 15 at 2,500 m: *p* = 0.007, *d* = 1.26, 95% CI = 0.17–1.24; medalists vs. Top 8 at 2,700 m: *p* = 0.027, *d* = 0.90, 95% CI = 0.06–1.29), with men staying in a group for longer (men medalists vs. Top 15 at 4,400 m: *p* = 0.013, *d* = 1.07, 95% CI = 0.30–2.88; medalists vs. Top 8 at 5,000 m: *p* = 0.004, *d* = 2.06, 95% CI = 0.61–3.67) (Figures 3, 4).

**Figure 6:**
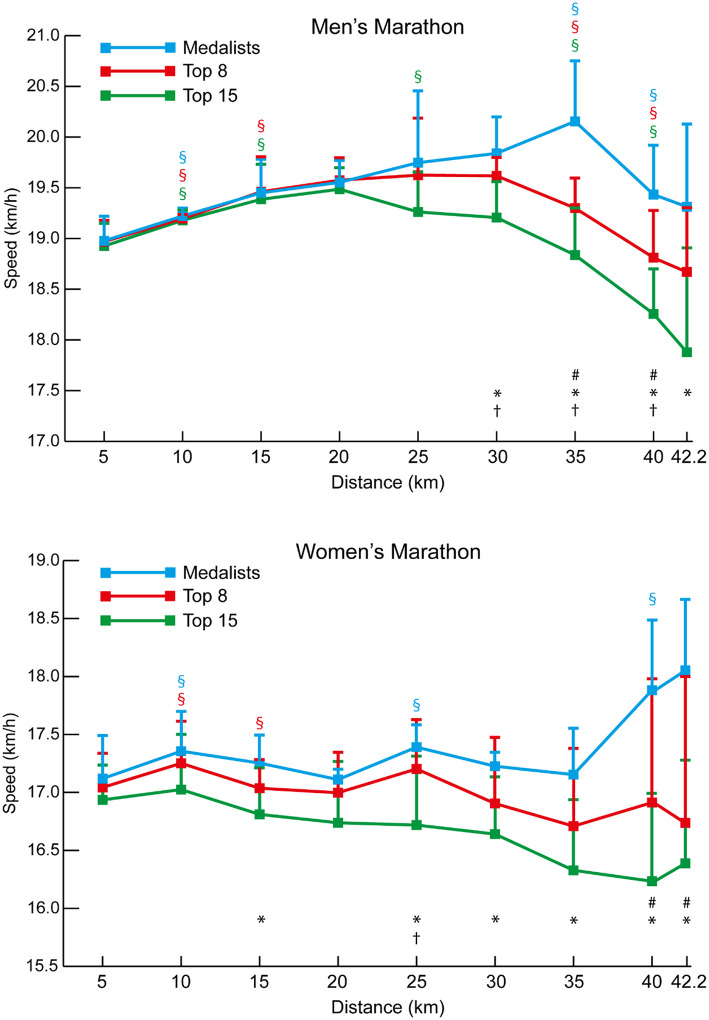
The mean (+ SD) 5-km and final 2.2-km segment speeds for each group of men and women marathon athletes. Differences between successive segments (*p* < 0.05, *d* ≥ 0.61) are represented by § (blue for medalists, red for Top 8, and green for Top 15). Differences in segment speed between groups (*p* < 0.05, *d* ≥ 0.61) have been annotated for medalists vs. Top 8 (#), medalists vs. Top 15 (*), and Top 8 vs. Top 15 (†).

**Figure 7:**
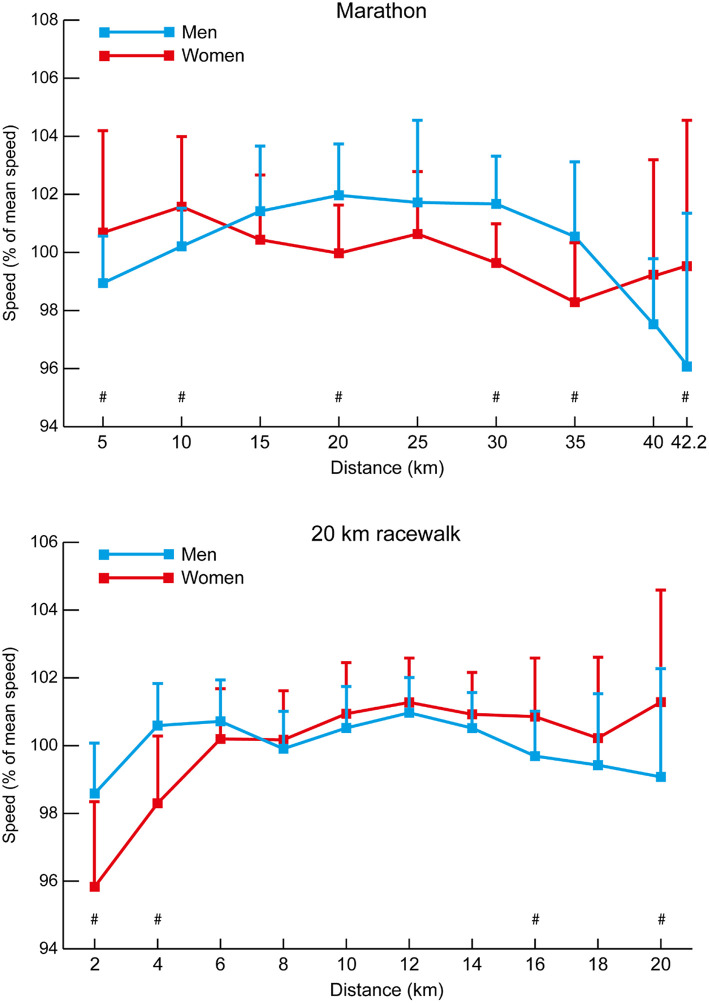
The mean (+ SD) section speed expressed as a percentage of mean speed for all men and women in the marathon and 20 km racewalk events. Differences in segment speed percentage between men and women (*p* < 0.05, *d* ≥ 0.61) have been annotated (#).

The mean speeds for each 5-km and final 2.2-km split for each group of marathon athletes show occasional and gradual increases in pace for the men until 35 km (10, 15, 25, 35 km: *p* ≤ 0.043, *d* = 0.65–1.51), whereas the women seemed to adopt more even pacing throughout (Figure 6), especially those in the Top 8 and Top 15 groups. All men medalists and eight of the nine women medalists ran negative splits. Seven of the 15 men and 10 of the 15 women in the Top 8 groups also ran negative splits, but only one of the 20 men and seven of the 21 women in the Top 15 groups managed negative splits. As in the track races, the separation of groups occurred earlier in the women's races (medalists vs. Top 15 at 15 km: *p* = 0.011, *d* = 1.08, 95% CI = 0.09–0.79; Top 8 vs. Top 15 at 25 km: *p* = 0.029, *d* = 0.83, 95% CI = 0.04–0.92). The mean 5-km and end 2.2-km segment speed percentages for all men and all women analyzed in the marathon events (Figure 7) show that the men started relatively slower than the women (at 5 km: *p* = 0.008, *d* = 0.68, 95% CI = 1.27–8.14), ran the middle section faster (at 20 km: *p* < 0.001, *d* = 1.16, 95% CI = 4.53–9.54), but then slowed more in the last sections (at 42.2 km: *p* = 0.001, *d* = 0.69, 95% CI = 3.13–12.04).

**Figure 8:**
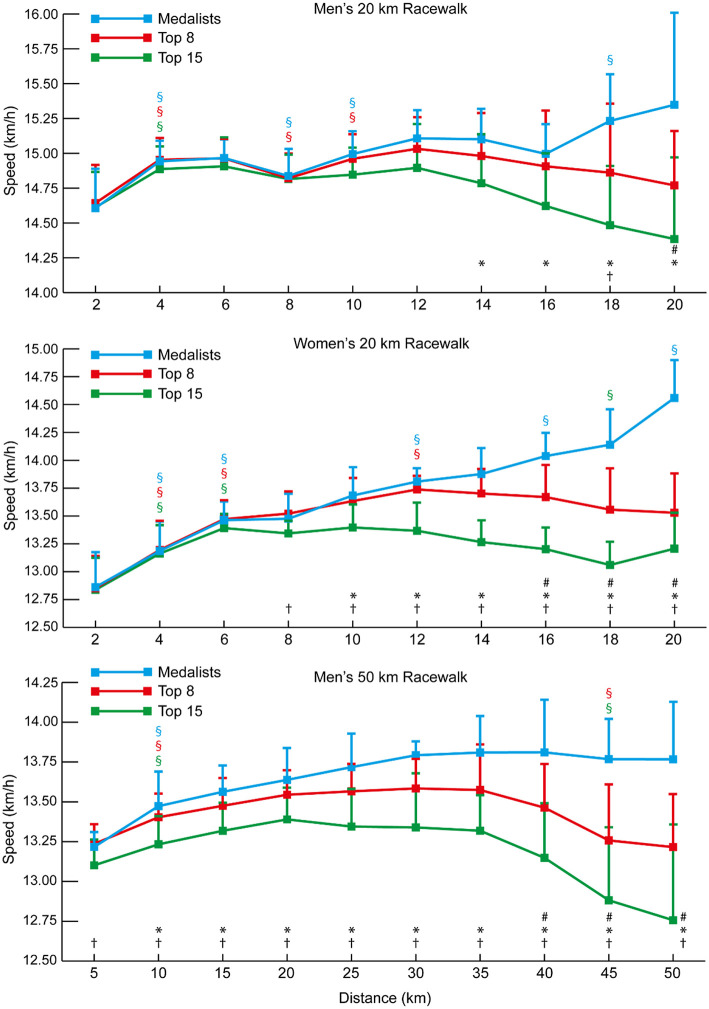
The mean (+ SD) 2-km segment speed (20 km event) and 5-km segment speed (50 km event) for each group of men and women racewalkers. Differences between successive segments (*p* < 0.05, *d* ≥ 0.61) are represented by § (blue for medalists, red for Top 8, and green for Top 15). Differences in segment speed between groups (*p* < 0.05, *d* ≥ 0.61) have been annotated for medalists vs. Top 8 (#), medalists vs. Top 15 (^*^), and Top 8 vs. Top 15 (†).

**Figure 9:**
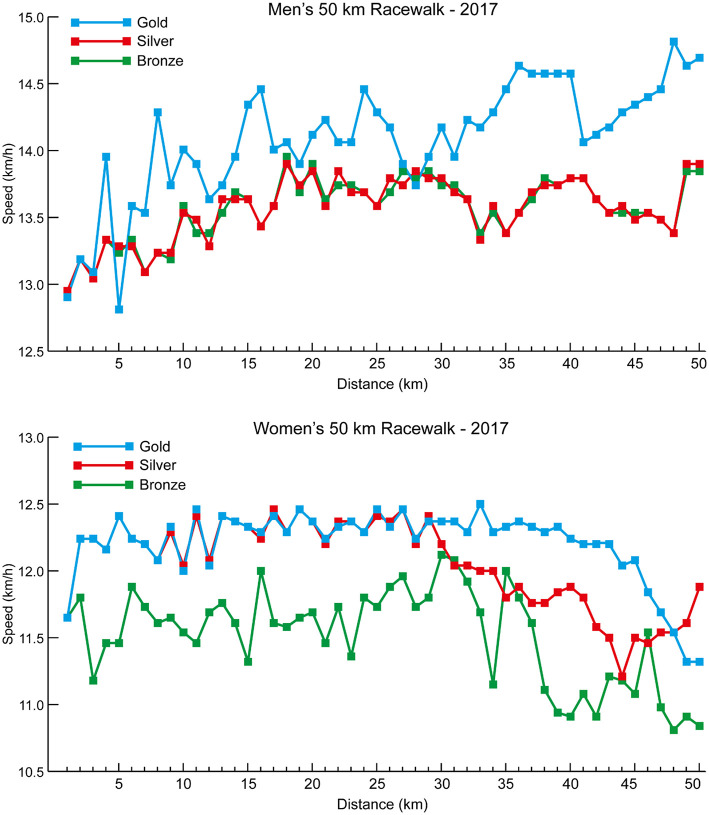
The individual 1-km segment speeds for the medalists in the men's and women's 50 km racewalk events at the 2017 IAAF World Championships.

The mean speeds for each 2-km split for each group of 20 km racewalkers (Figure 8) also show that the women's groups were separated earlier than in the men's race (women medalists vs. Top 15 at 10 km: *p* = 0.005, *d* = 1.27, 95% CI = 0.08–0.50; Top 8 vs. Top 15 at 8 km: *p* = 0.011, *d* = 1.12, 95% CI = 0.04–0.32), and that after 6–8 km the Top 15 group were unable to increase pace to keep up with the medalists and Top 8 athletes, with the medalists similarly being able to increase pace more so than the Top 8 as the race progressed. All medalists in both men's and women's 20 km racewalks achieved negative splits, as did 10 of the 15 men and 12 of the 15 women in the Top 8 groups. By contrast, only four of the 21 men and nine of the 21 women in the Top 15 groups racewalked negative splits. The mean 2-km segment speed percentages for all men and all women analyzed in the 20 km racewalks (Figure 7) shows that men started relatively faster (at 2 km: *p* < 0.001, *d* = 1.38, 95% CI = 3.99–7.77) and finished relatively slower (at 20 km: *p* = 0.002, *d* = 0.68, 95% CI = 2.27–9.75). The mean speeds for each 5-km split for each group of men's 50 km racewalkers (Figure 8) showed that the medalists were able to achieve near even paces for the whole race, whereas those finishing outside the medals slowed after 40 km (at 45 km: *p* ≤ 0.001, *d* = 0.65–0.66). In the men's 50 km racewalk, all medalists achieved negative splits, as did most (9 out of 15) of the Top 8 group. However, only six of the 21 men in the Top 15 group achieved negative splits. The individual pacing profiles of the medalists from the 2017 IAAF World Championships, shown in Figure 9, highlight the individual variation in pace during the 50 km racewalk.

## Discussion

Although elite athlete race data have previously been explored (Hanley and Hettinga, 2018; Hanley et al., 2019), this is the first study to examine high-resolution data sampling (i.e., sector times) of World Championship and Olympic pacing profiles across middle-distance, long-distance and racewalk events for men and women, while including for the first time data from the recently introduced 50 km women's racewalk event. The major insights from our study demonstrate the prevalent pacing strategies employed in the different IAAF events for men and women according to their finishing positions, while also showing for the first time distinct separation points in most events where the speed profiles for medalists start to follow a different trajectory from those of other competitors. The trajectories generally show that medalists are able to maintain high speeds throughout the entire race and are still able to speed up toward the end, whereas lower-finishing athletes are able to keep up with this medal pace for a period, but then tend to reach a point after which they slow down or are not able to accelerate as much as the medalists (Figures 1, 3, 4, 6, 8). In the 800 m event, this difference in trajectory emerges after 600 m and is a similarly critical point for both men and women, although men tend to have a faster start and slower finish (Figure 2). With 200 m remaining of the race, it is likely that high force metabolic fuel usage diminishes, and physiological conditioning is at its most discriminating influence (Fukuba and Whipp, 1999; Buchheit and Laursen, 2013). From this point, it appears that the athletes who slow down the least from the fast initial race pace are the most successful at achieving a medal performance. For the shorter distances (800 and 1,500 m), this happens toward the end of the race, characterizing them as more tactical (Renfree et al., 2014) in a head-to-head style of competition where the behavior of opponents plays a larger role. When athletes race in a pack or within each other's proximity for a large part of the race, the final stages have previously been shown to be decisive for winning (Konings et al., 2016a; Noorbergen et al., 2016) in other sports such as short track skating (Konings and Hettinga, 2018b). In our study, the longer IAAF distances such as the 5,000 m, 10,000 m, and 20 km racewalk demonstrate separation points earlier in the race and, particularly for the women, where there is only a small segment of the race where all competitors are together as a pack. This suggests competition outcomes are decided in the earlier stages of the race. In the women's marathon and men's 50 km racewalk, the pace differs between medalists and non-medalists from the start onward, changing the competition in terms of pacing and tactics more into an individual time-trial type events than head-to-head style competition. This inevitably means the dynamics of the competitive and tactical challenges in the race change with the style of event pacing (Konings and Hettinga, 2018d). In addition, it was notable in the marathon that men started slower than women, ran faster in the middle section, but then slowed more than women toward the finish (Figure 7). The pacing profiles in these championship marathons differed from those found in world record performances (Díaz et al., 2018, 2019) where pacemakers help the best athletes achieve even or negative pacing and highlights the need for athletes and coaches to appreciate the differences between championship and non-championship racing.

Interestingly, the separation by the medalists from those finishing in lower positions in most races occurs earlier for women than men, indicating that differences in the abilities of women competitors to maintain a high pace throughout the entire race are larger and deviations in pacing occur in the first part of the race. It is also possible the earlier separation of medalists from the racing pack for women could be due to tactical racing factors such as an early realization of being unable to sustain the required speed, or possibly because of greater variation in performance abilities among the runners in the race compared with men. For example, the winning margin between the gold and silver medalist in the 2017 women's 10,000 m was 46 s, whereas it was only 0.43 s in the men's event (IAAF, 2019); indeed, in this men's 10,000 m race the time difference between first and fifteenth athlete was less than the gap between first and second place in the women's race. However, Tables 1, 2 do not reveal greater variation among performances between men and women, indicating that our findings provide evidence that there could be greater earlier tactical awareness of appropriate pacing and performance limitations among women, similar to research on sex-based differences in running (Deaner et al., 2015).

The performances evaluated in this study provide useful insights into the strategies employed by finalists in IAAF and Olympic events and their relative success thus far. Men and women share many similar performance attributes and medal success is aligned with the same strategies for each race for both sexes. However, how that dynamically occurs in race pacing is quite different with much different trajectories of how races are managed. The earlier separation between groups for women is a novel finding that indicates some possible lines for future investigation exploring performance among both men and women. This thus results in different competitive and tactical challenges in women's races, that are more similar to individual competition, compared with the men, who run as a pack for longer. The pack running approach of men postpones the decisive stage of the race to the second half of the race in the 5,000 and 10,000 m races and thereby show profiles more similar to the classic head-to-head competition profiles as seen in short track skating (Konings et al., 2016a; Noorbergen et al., 2016). Interestingly, the individual analysis of medalists in the 50 km racewalk, using high-resolution 1-km splits for the first time, highlights these two different approaches. The winner of the men's 50 km adopted a time-trial approach, racing outside the pack and achieving an unprecedented 8-min winning margin. By contrast, the silver and bronze medalists, who were of the same nationality, adopted a pack approach where they raced side-by-side for almost the entire race (Figure 9). Adopting packing can be beneficial for performance but does lead to athletes using the exact same pace set by others, regardless of their personal optimal strategy (Hanley, 2015), and in the women's 50 km, the silver medalist could not stay in a pack with the leader. Athletes should note that different pacing profiles can arise in competition, especially over the longer distance races, and prepare for each eventuality in training.

It is evident that in the 800 m event, a positive pacing strategy was dominant for all groups of men and women, whereby the athletes ran the second lap slower than the first (Table 3), confirming pacing profiles identified previously for all rounds of 800 m championship racing (Hanley et al., 2019). The staggered start used in 800 m, which makes it unique amongst distance races, is a likely factor in this fast start given the athletes need to reach the 200-m distance in a strong tactical position, and do not have nearby opponents as pacing guides (Casado and Renfree, 2018; Hanley et al., 2019). It is not an unusual scenario whereby an 800 m runner performs poorly in a major championship, well below their season's best performance (Hanley et al., 2019), and this performance discrepancy can arise because the athlete has prepared for the championships by taking part in fast, structured races with pre-planned pacemakers. These non-championship races, such as those in the IAAF Diamond League, typically present a much more even paced profile (Filipas et al., 2018) than championship races, and middle-distance coaches should study carefully the actual pacing profiles that occur in 800 m championship racing and prepare their athletes accordingly. Beyond this race distance, the successful (i.e., medalist) strategy switches to a negative pacing profile, where speed is faster in the second half of the race. Indeed, for medalists, the strategic commonality is a wholly negative pacing profile for all distances beyond 800 m for all groups of men and women medalists (Table 3). Groups of athletes performing slower than the medalists (e.g., Top 8 and Top 12/15) show greater variation in pacing strategies with some adopting positive pacing profiles whereby pace declined in the second half of the race in contrast to the highest performing athletes. Nevertheless, medalist success in all events was consistently achieved with a negative pacing strategy for men and women in events beyond 800 m. There are several possible causes for a negative pacing strategy in distance events, but most likely it reflects the small margins of difference in performance times between athletes (Table 1) competing in head-to-head competition, and the necessity when racing to finish an event strongly to win among equally motivated and similarly capable opponents. The impact of opponents has recently been explored in research that clearly demonstrates that racing poses a tactical challenge to all competitors (Konings and Hettinga, 2018b), and in athletics it has been demonstrated that some retention of physical reserve is important to be able to respond to the strategies of opponents (Mytton et al., 2015).

In the longer events, a similar pattern emerges for all distances, although, as these are performed with a negative pacing strategy, pace becomes faster in some cases. Rather than slowing down the least being most important (as in the 800 m), the medalists emerge on a different pacing trajectory. It is interesting that the crucial point of separation in pacing trajectory occurs at a much earlier stage of races for women than men in events longer than 800 m. For example, in the 1,500 m (Figure 1), male medalists separated from the slowest runners (those finishing 9th−12th) and the Top 8 athletes in the last 100 m. This same effect can be seen for women at 1,200 m for the Top 12 athletes and 1,300 m for the Top 8. Similarly, over 5,000 m, male medalists separated from the slowest pack members later (84% of race distance: 4,200 m) than for women, for whom this occurred at 50% of race distance (2,500 m). Clear trajectories emerge for those finishing as medalists vs. middle-ranked non-medalists and the slower remaining athletes. In the 10,000 m race, the effect is very pronounced (Figure 4) with men remaining as a tighter group up to 80% of distance where the slower athletes are dropped from the pack, and the Top 8 athletes are only dropped at 96% of total distance. For women, the slowest pack begin to run slower at only 17% of race distance, with the Top 8 being dropped at 53% of race distance. Such profiles and patterns can be seen across all events up to and including the 50 km racewalk. It should be noted, however, that in many events the medalists did not run faster than lower-finishing athletes in every split after a certain distance, and thus these small bursts of speed gradually separated the best athletes from the others until the gap between them was too large to overcome (Fukuba and Whipp, 1999). Like the fast start over the first 200 m in the 800 m, the lack of a difference in split speeds can be due to tactical approaches, such as only overtaking on the straight 100-m sections (Aragón et al., 2015). A key point shown by the current study is that in championship racing, an even pace is not necessarily a winning pace; the ability to keep energy in reserve for a fast endspurt or smaller, but sustained bursts throughout the race is important for separating from lower-placed finishers and might be developed by the high proportion of tempo running and short interval training sessions adopted by world-class distance runners and are thus recommended to coaches (Casado et al., 2019).

Decreases in running speed are not always directly related to metabolic fatigue (Renfree and St Clair Gibson, 2013); other reasons for disparities in pacing strategy between the successful negative pacing strategy employed by medalists and positive pacing (i.e., slower in the second half of a race) by lower performing athletes could be a psychological acceptance of the inevitability of race outcome by the non-medalists. It is plausible that once an approximate order of supremacy has been established in a race, lower performing athletes who are not in contention for a medal accept the race outcome to some extent, retain some energy and do not increase their pace to that commensurate with maximal effort. This tactic can be adopted by lower-finishing athletes to avoid a “catastrophic event” (Thiel et al., 2012), whereby the athlete would run so fast relative to ability in trying to keep up with the leaders that they would have to drop out. In events such as rowing, it is common to see differential pacing strategies according to ability in a race, where race outcome is often established as early as 25% of the total distance (Edwards et al., 2016) and ~50% of distance in Olympic events (Garland, 2005). However, it should not be forgotten that many athletes are likely to be realistic about their prospects in global finals and set goals relative to their ability: setting a personal best time, making the final, or finishing in the top 8 or top 15 can be important for an individual athlete, especially as finishing in these positions can be linked to future governing body funding (e.g., Athletics Ireland, 2019), and success is a relative term.

It is possible that our findings simply provide evidence that lower performing athletes in races adopt an overly optimistic initial race pace to match the pace of the eventual medalists for as long as they can, and the consequence of this is to experience progressively greater accumulation of fatigue and a slowing of pace over the race distance compared with that of medalists (Renfree and St Clair Gibson, 2013). This tactic of following the leader is the least psychologically taxing strategy given the lack of conscious pace judgment but can mislead less able athletes into adopting an unsustainable pace (Renfree et al., 2015; Hanley, 2018). The metabolic cost of sustaining a common fixed running speed is less physically demanding for the highest performance athletes than slower athletes because the intensity is at a lower percentage of their maximum capabilities (Filipas et al., 2019). As such, it is not surprising that lower performing athletes have less available energy later in a race to adopt the same racing strategy as the medalists. Certainly, in comparison with a time trial, a race is characterized by attempting to beat the opposition rather than focusing purely on performance time, and other competitors influence the process of decision-making regarding how and when to invest energy over a race (Konings et al., 2016b; Konings and Hettinga, 2018a). This can lead to dynamic and tactical pacing in a race situation to which athletes are unaccustomed, and which is thought to increase the physical challenge of exercise compared with self-paced exercise (Lander et al., 2009). This can manifest as misjudgment of initial race pace among the lower performing athletes or a willingness to match the pace of the medalists where the consequences are eventually being unable or unwilling to sustain a higher than usual pace, and thereafter suffering the consequences of fatigue and diminishing pace. Once the slower athletes realize they can no longer proceed at the same pace as the faster athletes, separation from the racing pack occurs and acceptance can set in thus lowering their pace further, adding to the performance costs of having already run the race to this stage at a speed faster than they might have otherwise planned. It has been shown that dependency of the opponent impacts on decision-making, so when athletes are dependent of the other athlete to win, this changes competition dynamics. A higher interdependency between athlete and opponent alters in-race adaptations based on the opponent's behavior (Konings et al., 2019). In the context of exercise regulation, attentional cues such as the proximity of the opponent are likely to be used in an adaptive way according to their availability and situational relevance, consistent with a decision-making framework based on the interdependence of perception and action. However, this raises the question of whether non-medalists might perform better if they ignored the pace of runners showing faster initial pace in the race if it leads to misjudgment in their own performance and premature fatigue. They might simply do better to complete their own race strategy in isolation, and adopt an even pace, as occurs in non-championship races (Filipas et al., 2018). Yet, it might also be that winning chances are only present when in the leading group, so even though deciding to race one's own pace could lead to better performance, athletes might not want to give up their chance to win, particularly in highly competitive sports environments (Casado and Renfree, 2018; Hanley and Hettinga, 2018) with marathon runners, for example, being prepared to risk a potentially harmful fast start if it presents them with the chance of success (Deaner et al., 2019). The impact of high motivation and passion for sports has previously been linked to athletic decision-making within a race but also throughout a season (Schiphof-Godart and Hettinga, 2017) and is expected to be particularly relevant in high-standard head-to-head events such as the IAAF World Championships.

The performance implications of pacing in the heat such as in the 2019 IAAF World Championships in Doha or Olympic Games in Tokyo could require considerable adjustment in pacing strategy. Exercise in the heat poses severe challenges that mean either the same strategic approach to racing or the adoption of a different strategy. Heat often proves a decisive factor in performance outcome, particularly in endurance events (Guy et al., 2015). For example, an earlier study of race outcomes in IAAF World Championships (1999–2011) (Guy et al., 2015) demonstrated that in hot environments (>25°C), endurance performances were worse (~3% reduction in performance, Cohen's *d* = 0.8; large impairment), compared with cooler conditions (<25°C). By contrast, performance in short duration sprint events was augmented in the heat compared with temperate conditions (~1% improvement, Cohen's *d* = 0.8; large performance gain). Consequently, understanding the demands of the race, the opposition and the environmental challenges of racing in the heat will all be crucial to success. The heat effects of Doha's climate are most likely to affect the road races (marathon and racewalks) (Ely et al., 2008) as these will be held outside the air-conditioned stadium. To account for the likely hot conditions, the road races will be held at nighttime (approximately midnight) and this in itself could present challenges for athletes more accustomed to competing in the early morning or evening. Although it is possible, it seems unlikely that negative pacing in endurance events would not be adopted in the heat given its consistent success, and therefore a within-style slowing of pace adjustment is probably to cope with the environmental and course conditions (Angus, 2014). Nevertheless, further high frequency analysis of elite performance pacing in the heat would be a meaningful contribution to the literature when sufficient data become available.

Analysis of such comprehensive data as explored in the present study provides an opportunity to investigate tactics and race strategies pertinent to current race performances for those men and women currently competing in world-class distance events, providing meaningful insights for upcoming events. Indeed, the results found provide an invaluable guide for coaches to prepare their athletes for the likely pacing profiles adopted in each of these events, even though a limitation of the current study design is that analyses are purely descriptive and have not taken place under controlled circumstances, which means that findings and potential extrapolations to other races need to be interpreted with care. Confounding factors could be, for example, differences in climates, altitude and timing of the events. However, at the same time, our descriptive analysis of in-race data of world-class athletes is highly ecologically valid, and relies on a very large database with high resolution performance analysis, which is a strength of the current approach and could provide meaningful and unique insights into world-class competition. In analyzing the world's best athletes, it was also inevitable that particularly successful athletes were analyzed in more than one competition. On the one hand, this means that caution must be taken when interpreting the statistical results, but on the other it does allow for an appreciation of how contemporary successful athletes pace their races.

## Conclusions

This is the first study to demonstrate a comprehensive separation effect for pacing by medalling athletes and their opponents across the full range of middle-distance, long-distance and racewalk events for men and women. This effect seems consistent for men and women, although it occurs at an earlier stage of most race distances for women. However, it is also evident in events such as the marathon and racewalks, particularly among women, that the pacing trajectories of medalists vs. other athletes can take completely different paths from the start of the race, indicating the specific impact of opponents on each race distance is likely to be different across race distances. As such, some races might be less about direct impacts of head-to-head competition than being more akin to contested time-trial performances. It is also evident that, in events longer than 800 m, the commonly successful racing strategy is to complete the first half of the race at a slower pace than the second half (negative pacing). This demonstrates the importance of the ability to change speed and respond dynamically to changes in pace in endurance events although the dominant characteristic of long-distance athletes is endurance capability.

## Data Availability

The datasets generated for this study are available on request to the corresponding author.

## Ethics Statement

Ethical review and approval was not required for the study on human participants in accordance with the local legislation and institutional requirements. Written informed consent for participation was not required for this study in accordance with the national legislation and the institutional requirements.

## Author Contributions

BH conducted data analysis and graphical representation of the findings. All authors contributed to conception and design of the work, drafted it, and revised it critically for important intellectual content, approved the final version of the manuscript, agree to be accountable for all aspects of the work in ensuring that questions related to the accuracy or integrity of any part of the work are appropriately investigated and resolved. All persons designated as authors qualify for authorship, and all those who qualify for authorship are listed.

### Conflict of Interest Statement

The authors declare that the research was conducted in the absence of any commercial or financial relationships that could be construed as a potential conflict of interest.
